# Diversity and ecological functions of anthocyanins

**DOI:** 10.1186/s12870-025-08006-3

**Published:** 2025-12-23

**Authors:** Nele Grünig, Jakob Maximilian Horz, Boas Pucker

**Affiliations:** 1 Plant Biotechnology and Bioinformatics, Institute of Plant Biology & BRICS, TU Braunschweig, Mendelssohnstrasse 4, Braunschweig, 38106 Germany; 2 Institute for Process Engineering in Life Sciences, Karlsruhe Institute of Technology (KIT), Kaiserstraße 12, Karlsruhe, 76131 Germany; 3 Institute for Cellular and Molecular Botany (IZMB), University of Bonn, Kirschallee 1, Bonn, 53115 Germany

**Keywords:** Anthocyanins, Flavonoids, Photoprotection, Antioxidants, Pollination, Coloration, Transcriptional regulation, Intracellular transport

## Abstract

Anthocyanins are well known as colorants of flowers, but many other invisible functions might have been more important during the evolution of complex biosynthesis networks in plants. The number of anthocyanin decorating enzymes, the subtle control of structural genes by numerous transcription factors, and routes of intracellular anthocyanin transport are reviewed here. Various ecological functions of anthocyanins hold the key to understanding evolutionary trajectories that lead to the success of these pigments. Proposed functions include carbon sinks that prevent excessive sugar levels, sun blockers protecting the photosynthesis apparatus, antioxidants scavenging reactive oxygen species, providing camouflage, and attraction of pollinators and seed dispersers. Anthocyanins clearly fulfill different functions in different plant organs. It is currently believed that protective functions in leaves gave rise to the biosynthesis network and that flower and fruit coloration evolved later. Despite decades of research on the anthocyanin biosynthesis, there is still substantial potential for fundamental discoveries.

## Background

Anthocyanins are well known as a phylogenetically widespread group of plant pigments providing colorful patterns to flowers and fruits. Research on anthocyanins has a rich history of interdisciplinary explorations dating back more than 100 years [1]. Anthocyanins can provide a range of different colors including red, orange, pink, purple, and blue. This turns anthocyanins into an economically relevant target in the engineering of horticultural plants [2, 3]. Especially the rise of genome editing methods like CRISPR/Cas in plants [4] opens new avenues for targeted modification of the anthocyanin biosynthesis to achieve a desired flower color. However, such endeavors require a comprehensive understanding of all genes involved in the biosynthesis pathway. Due to the clearly visible phenotype, the anthocyanin biosynthesis quickly emerged as a model system for research on biosynthesis pathways and has even been described as a metamodel for understanding the genetic basis of evolutionary change [5, 6]. First studies investigating genes of the anthocyanin biosynthesis date back to the 1950s, when Barbara McClintock studied transposons in maize [7] that generated diverse pigmentation patterns by disrupting anthocyanin biosynthesis genes. First biochemical studies of anthocyanins in flowers are even older - ranging at least back to 1835 [8]. Anthocyanins were also involved in classical genetic experiments performed by Gregor Mendel, as he worked on the flower color of peas [9]. However, it took almost 150 years until the underlying genes were discovered [10, 11]. Anthocyanins are relevant for studies in many different fields, because they are present in almost all plant lineages. However, a recent, transcontinental study revealed that only about 56% of 926 analyzed animal-pollinated species from California, Southern Spain, and Southeastern Brazil have floral anthocyanins [12]. Given this distribution, it is not surprising that numerous studies described the loss of anthocyanin pigmentation in flowers due to mutations [13–16]. This lack of pigmentation in individual plants, at a species level, or at the genus level can be identified visibly and thus received substantial attention in ecological and evolutionary studies [16, 17]. In many cases, not the anthocyanin biosynthesis genes themselves are mutated, but changes in their transcriptional regulators prevent an activation of the anthocyanin biosynthesis apparatus [16]. A noteworthy exception to the almost ubiquitous distribution of anthocyanins is the flowering plant order Caryophyllales, in which anthocyanins have been replaced by betalains, another pigment type with partial functional redundancy [18]. Anthocyanins and betalains appear mutually exclusive as some families within the Caryophyllales maintained their anthocyanin pigmentation, while others show only betalain pigmentation [18, 19]. This complex pattern of pigment biosynthesis emergence and loss in the Caryophyllales provides an excellent system for evolutionary studies on biosynthesis pathways [20, 21]. Recently, two additional major loss events of the canonical anthocyanin biosynthesis have been reported at the family level in the Poaceae and Cucurbitaceae [22, 23]. The Poaceae regained the ability to produce anthocyanins through independent evolution of an anthocyanin-related glutathione-S-transferase [22]. The Cucurbitaceae might represent the first example of anthocyanin replacement by carotenoids [23]. To fully understand why anthocyanins have been replaced by other pigments or why anthocyanin loss might be neutral or even advantageous under specific conditions, it is important to first clarify the biochemical and ecological functions of anthocyanins. This is a challenging undertaking given the biochemical diversity of anthocyanins facilitated by species-specific differences in the anthocyanin biosynthesis pathway and promiscuous enzymes that form a complex network of interconnected pathways.

In this review, we will summarize the existing knowledge about the biosynthesis of anthocyanins, the transcriptional control of this biosynthesis pathway, the intracellular transport of anthocyanins, and their diverse ecological functions. 

### Biosynthesis of a diverse set of anthocyanins

**Fig. 1:**
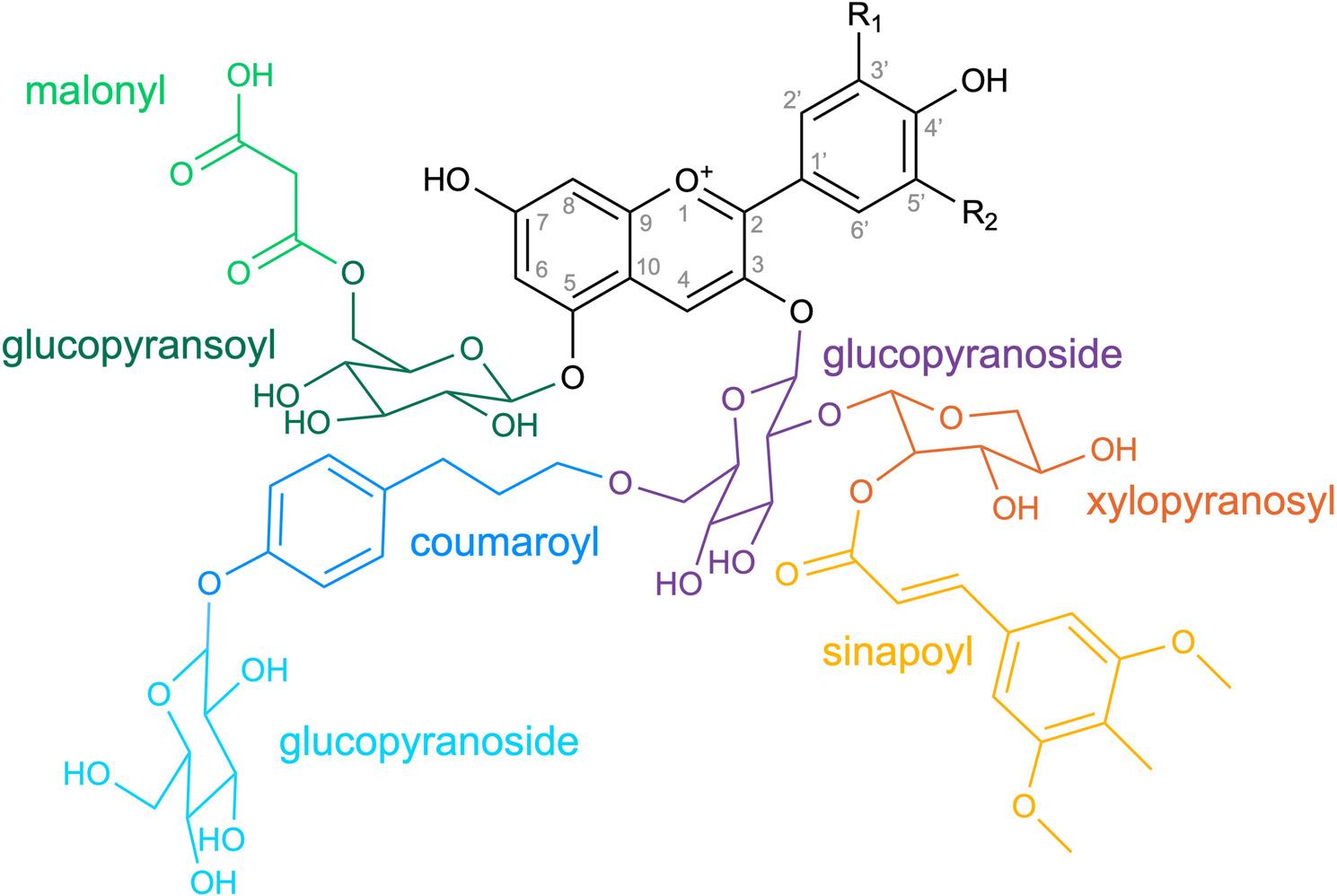
A11 (cyanidin 3-O-[2-O(2-O-(sinapoyl)-β-D-xylopyranosyl) 6-O-(4-O-(β-D-glucopyranosyl)-p-coumaroyl-β-D-glucopyranoside] 5-O-[6-O-(malonyl) β-D-gluco-pyranoside]) is the major anthocyanin in the leaves and stems of *Arabidopsis thaliana* [29, 30]. The IUPAC name was retrieved from PubChem [31]. The visualization of stereochemistry might deviate slightly due to spatial organization

Anthocyanins comprise an aglycone that can be decorated by a range of sugar moieties, acids, and methyl groups (Fig. 1). While the biosynthesis of the aglycone is well understood and largely conserved between plant species, the decoration is highly diverse and often differs between species or larger evolutionary lineages [24]. For example, cyanidin 3,7,3’-triglucosides appear to be specific to *Epidendroideae* [25]. Flavonoid patterns are even considered useful characters in phylogenetic studies [26]. A general dominance of glucose in the glycosylation of anthocyanins has been reported [27] and acetyl, malonyl, malyl, and succinyl appeared to be the most abundant acyl moieties [28]. The current knowledge about genes involved in the decoration of anthocyanins is limited to a small number of plant species. The huge diversity of flower colors and hues seen in other parts of plants suggest an enormous variety of different anthocyanin derivatives and combinations of these. Therefore, it is likely that most anthocyanin modification reactions have not been discovered and characterized yet.

### Anthocyanin biosynthesis as a branch in the flavonoid biosynthesis

**Fig. 2:**
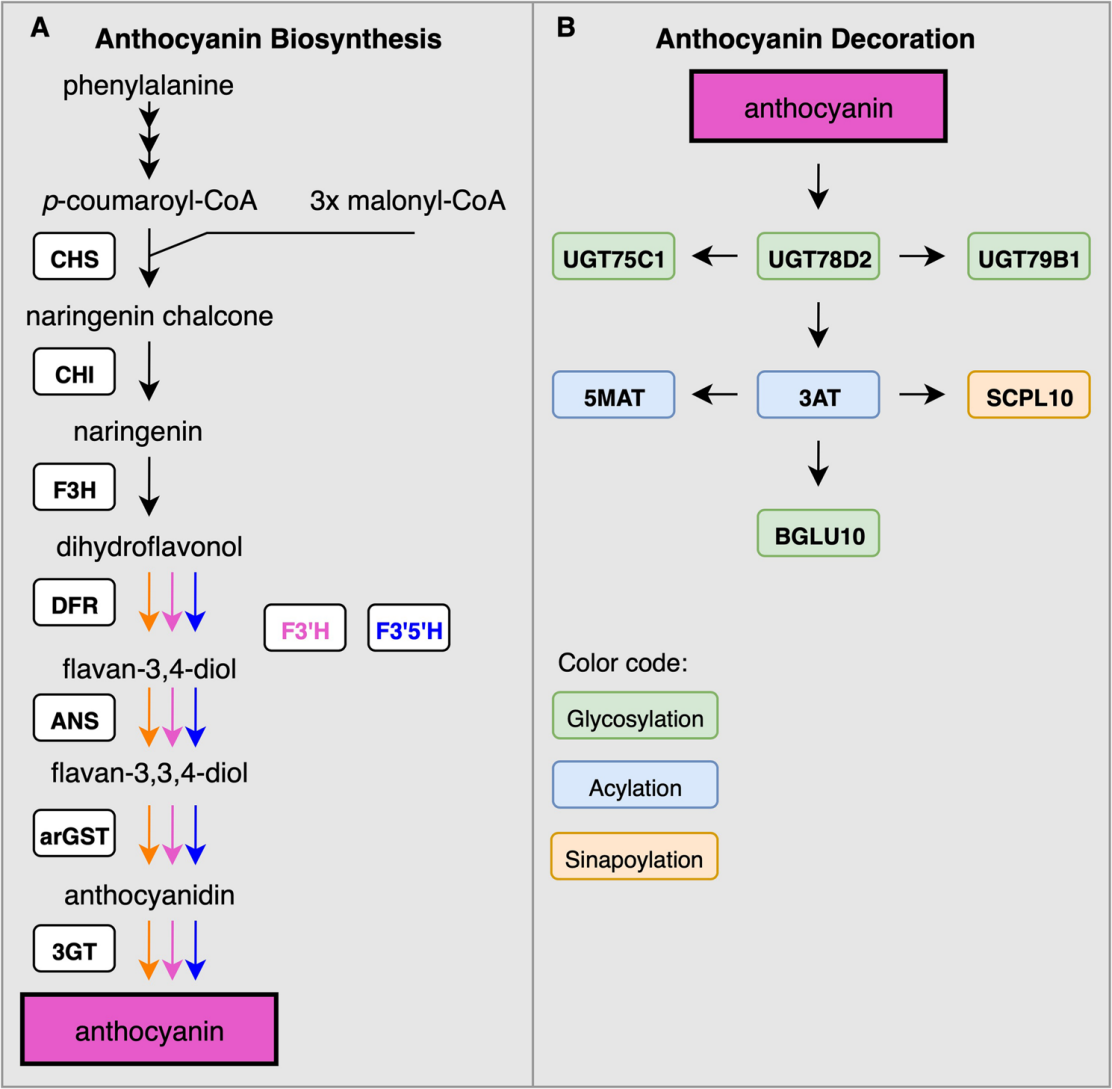
Simplified illustration of the flavonoid biosynthesis with focus on the anthocyanin branch (**A**). Enzymes involved in the modification of anthocyanins as reported in *A. thaliana* (**B**). Displayed enzymes are chalcone synthase (CHS), chalcone isomerase (CHI), flavanone 3-hydroxylase (F3H), flavonoid 3’-hydroxylase (F3’H), flavonoid 3’,5’-hydroxylase (F3’5’H), dihydroflavonol 4-reductase (DFR), anthocyanidin synthase (ANS), anthocyanin-related glutathione S-transferase (arGST), UDP-dependent anthocyanidin-3-O-glycosyltransferase (3GT), UDP-glucosyltransferase 75C1 (UGT75C1), UDP-glucosyltransferase 78D2 (UGT78D2), UDP-glucosyltransferase 79B1 (UGT79B1), Malonyl-CoA:anthocyanidin 5-O-glucoside-6''-O-malonyltransferase (5MAT), Coumaroyl-CoA:anthocyanidin 3-O-glucoside-6''-O-coumaroyltransferase (3AT), sinapoyl-β-D-glucose:anthocyanin sinapoyl transferase 10 (SCPL10), and β-glucosidase 10 (BGLU10)

Anthocyanins are produced through one branch of the flavonoid biosynthesis pathway (Fig. 2). Other branches of the flavonoid biosynthesis are competing for substrate with the anthocyanin biosynthesis and lead to biochemically different subclasses of flavonoids including flavones, flavonols, or proanthocyanidins. Phenylalanine is considered as the substrate of the anthocyanin biosynthesis. The general phenylpropanoid pathway comprising the three enzymes phenylalanine ammonium lyase (PAL), cinnamate 4-hydroxylase (C4H), and 4-coumarate: CoA ligase (4CL) processes phenylalanine and provides p-coumaroyl-CoA as substrate to the flavonoid biosynthesis. The enzymes naringenin-chalcone synthase (CHS), chalcone isomerase (CHI), flavanone 3-hydroxylase (F3H), dihydroflavonol 4-reductase (DFR), anthocyanidin synthase (ANS), and anthocyanin-related glutathione S-transferase (arGST) produce anthocyanidins which can be converted into anthocyanins through the addition of a sugar moiety by an UDP-dependent anthocyanidin-3-O-glucosyltransferase (3GT) [5, 32, 33]. The diversity of anthocyanins produced through this pathway is increased by two enzymes that can add additional hydroxyl groups to the B ring of the molecule: flavonoid 3’-hydroxylase (F3’H) and flavonoid 3’,5’-hydroxylase (F3’5’H) [34]. With increasing numbers of hydroxyl groups on the B-ring, anthocyanins are classified as pelargonidin, cyanidin, or delphinidin derivatives. This hydroxyl group difference has important functional consequences, because the color of the molecules ranges from orange-red (pelargonidin derivatives) to blue (delphinidin derivatives). Furthermore, the hydroxylation pattern influences the antioxidant capacity of the anthocyanin [35]. Since genes required for this anthocyanin core biosynthesis are well conserved across plant species, their identification based on orthology is straightforward [24, 36] and resulted in a large number of studies reporting on just these genes in numerous species [16].

### Enzyme promiscuity associated with the anthocyanin biosynthesis

Catalytic promiscuity is a feature of the specialized plant metabolism that can help to explain the plethora of different metabolites produced by a comparatively small number of enzymes and forms the basis for the evolution of novel enzymes [37]. One prominent example in the flavonoid biosynthesis is the first committed enzyme CHS, which catalyzes the production of naringenin chalcone, but also has the ability to produce other polyketides [38, 39]. The binding of CHI-like (CHIL) proteins to CHS rectifies this promiscuity and ensures the efficient production of precursors for the following step in the flavonoid biosynthesis, but does not substantially increase the activity of CHS as initially assumed [38–41]. CHS duplication and subsequent loss of CHIL interaction ability resulted in the evolution of novel enzyme functions through neofunctionalization [38]. The interaction of CHS and CHIL appears conserved across all land plants and CHILs show species-specific affinity for CHS [38]. Another striking example of catalytic promiscuity is provided by members of the large 2-oxoglutarate-dependent dioxygenase family that are active in the flavonoid biosynthesis: F3H, flavonol synthase (FLS), and ANS [42–47]. While these enzymes predominantly catalyze different reactions, they can partially catalyze each other’s reaction with the extent of these side activities appearing species-specific [42–46]. Gene duplication followed by neofunctionalization has been identified as an important factor contributing to the metabolic diversity across species [48]. In the Apiaceae, *FNS* I was identified as another member of this gene family that evolved through gene duplication from *F3H* [43, 49]. However, F3H is assumed to have evolved from a promiscuous ancestor that harbored flavone synthase activity [45]. In summary, genes encoding promiscuous enzymes that undergo specialization after gene duplications appear to be a predominant theme in the evolution of the flavonoid biosynthesis.

### Diversity of anthocyanin decoration

Genes associated with the decoration of anthocyanins are less conserved and thus knowledge about them beyond *A. thaliana* is sparse in the literature. Studies in *A. thaliana* identified a range of genes that are associated with the anthocyanin decoration including *UGT75C1*/At4g14090 [30], *UGT79B1*/At5g54060 [50], *3AT1*/At1g03940 and *3AT2*/At1G03495 [51], *BGLU10*/At4g27830 [52], *5MAT*/At3g29590 [51], and *SCPL10*/At2g23000 [53]. Glycosylation of anthocyanidins constitutes the final step of the anthocyanin biosynthesis. Glucosyltransferases can be classified based on the source of the sugar moiety: UDP-glucose or acyl-glucose. Only the UDP-dependent glycosyltransferases appear to be relevant enzymes for the transfer of other sugar moieties besides glucose. Usually, anthocyanidins are first glycosylated at the C3 position. Tohge *et al* (2005) demonstrated that UGT78D2 (At5g17050) catalyzes this glycosylation of anthocyanidins at the C3 positions. Like many other UGTs, UGT78D2 also shows a high substrate promiscuity: In *A. thaliana* and *Vitis vinifera*, 3-O-glycosylation of flavonols is also catalyzed by UGT78D2 [30, 54]. In *Glycine max*, *Gm*UGT78K1 from black seed coat showed 3-O-glycosylation activity of anthocyanidins and flavonols [55]. Furthermore, UGT78G1 (GT83F) was identified as another enzyme that can perform 3-O-glycosylation of pelargonidin and cyanidin, while mostly acting on other flavonoids [56, 57]. Interestingly, this enzyme has been reported to also catalyze deglycosylation of anthocyanidin 3-O-glycosides, i.e., the removal of a sugar moiety from an anthocyanin [57]. Following the 3-O-glycosylation, an additional 5-O-glycosylation is possible. Several studies reported 5-O-glycosyltransferases in a range of plant species. The best studied UDP-glycosyltransferase adding a glucose at the 5-position of the anthocyanin A-ring is the *A. thaliana* UGT75C1/At4g14090 [30]. UGT75C1 is responsible for glycosylation of anthocyanidin 3-O-β-D-glucosides leading to a 3-O-5-O-diglucoside product. Anthocyanidin 3-O-5-O-diglucosides have been reported to be more stable and soluble, responsible for bright-purple flower coloration [58] and form the basis for further complex modifications, e.g. sinapoylation or coumaroylation. Multiple UDP-dependent 5GTs were identified in *Dahlia variabilis* [59], *Petunia hybrida* [60], and *Gentiana triflora* [61]. Nakatsuka *et al* (2008) showed that the *Gt*5GT accepts different anthocyanidin 3-O-beta-D-glucosides as substrates whereas the enzyme does not show activity using aglyconic anthocyanidins as substrates [61]. As reported for multiple UGTs, UGT75C1 also has a high promiscuity. *Sl*UGT75C1 glycosylates abscisic acid (ABA) and IAA [62]. This example shows additional roles of UGT75C1 in fruit ripening and drought resistance in *Solanum lycopersicum* thus supporting a broad substrate promiscuity even beyond anthocyanins. Despite this high promiscuity, *At*UGT75C1 does not appear to participate noticeably in flavonol glycosylation [63]. To the best of our knowledge, there are no reports about UDP-dependent enzymes catalyzing a 7-O-glycosylation of anthocyanins. However, *Dg*AA7GT from *Delphinium grandiflorum*, an acyl-glucose-dependent glycosyltransferase, is proposed to glycosylate anthocyanidin 3-O-glycosides and anthocyanidin 3-O-malylglycosides [64]. In monocotyledones, *Aa*AA7GT from *Agapanthus africanus* was found to 7-O-glycosylate anthocyanidin 3-O-glycosides, 3-O-galactosides and 3-O-rutinosides [65]. Both AA7GTs do not accept aglyconic anthocyanidins as substrates. Various galactosylated anthocyanidins were reported in different *Actinidia* species: cyanidin- and delphinidin 3-O-galactosides, as well as cyanidin- and delphinidin 3-[2-(xylosyl)galactosides], requiring the activity of galactosyltransferases [66]. In *Vigna mungo*, an UF3GaT was described, showing high UDP-dependent 3-O-galactosylation activity for different flavonols and anthocyanidins [67]. UCGalT1, also an UDP-dependent galactosyltransferase, was identified in purple carrots (*Daucus carota)* taproots and purple celery (*Apium graveolens)* [68, 69]. Recombinant expression of *DcUCGalT1* as well as *AgUCGalT1* in *Escherichia coli* led to the presence of 3-O-galactosidated anthocyanins. Neither enzyme showed activity with UDP-glucose or UDP-xylose as a sugar donor but had a high promiscuity regarding the sugar acceptor: Both UCGalT1 showed activity when incubated with flavonols (quercetin and kaempferol) and UDP-galactose, whereas *Dc*UCGalT1 also catalyzed the galactose transfer of UDP-galactose to cyanidin, peonidin and pelargonidin [68, 69].

3-O-glycosylation is the prerequisite for multiple further modification steps. The addition of another sugar to the 3-O-glycosyl residue of the anthocyanin leads to formation of disaccharide residues, e.g. the attachment of rhamnose to 3-O-glycosylated anthocyanins results in anthocyanin-rutinosides [70]. Multiple anthocyanin rutinosides in different plant species, especially *Petunia* and *Solanum*, were detected [27]. Among others, 6-hydroxycyanidin 3-rutinoside, cyanidin 3-rutinoside, pelargonidin 3-rutinoside were found in *Alstroemeria* cultivars [71]. The first rhamnosyltranserases were described in *Silene dioica* and *Petunia*, catalyzing the addition of a rhamnosyl group to 3-O-glycosylated and 3,5-diglycosylated anthocyanins [72–74]. In 2013, *Cs1*,*6RhaT*, encoding for an anthocyanin 3-O-glycoside 1,6-rhamnosyltransferase, was discovered, catalyzing the formation of peonidin- and cyanidin rutinosides in *Citrus* species with a high promiscuity, showing affinity for flavanones, flavones, and flavonols [75]. Interestingly, the gene is only common to non-bitter *Citrus* species, whereas bitter-tasting *Citrus* species have the gene *Cm1*,*2RhaT*, encoding a 1,2-rhamnosyltransferase that rhamnosylates flavanone-7-O-glycosides [76]. In *Lobelia erinus*, two UDP-dependent rhamnosyltransferases were found (ABRT2 and ABRT4) to rhamnosylate anthocyanin 3-O-glycosides [77].

In contrast to UDP-dependent glycosylation, AAGTs are acyl-glucose-dependent anthocyanin glucosyltransferases belonging to the glycoside hydrolase family 1 (GH1) [64]. Multiple BGLUs are shown to be responsible for encoding GH1-type glycosyltransferases using already substituted flavonoids as a substrate in *A. thaliana* [52, 78, 79]. However, only *AtBGLU10* seems to play a role in anthocyanin modification [52, 78], whereas BGLU1, BGLU3, BGLU4, and BGLU6 prefer flavonols over anthocyanins as substrates [79, 80].

The most common anthocyanins have a xylosyl-group attached to the 2’’-position of the 3-O-sugar. In *A. thaliana*, *At*UGT79B1 is known to catalyze the sugar attachment to 3-O-glycosylated anthocyanins [50]. Different anthocyanins can act as sugar acceptors, i.e. cyanidin 3-O-glucoside, delphinidin 3-O-glucoside and pelargonidin 3-O-glucoside. Notably, no activity was observed with cyanidin 3-O-rhamnoside, whereas activity was detected when cyanidin 3-O-rhamnosyl(1→6)glucoside was used as the sugar donor [50]. The enzyme also has a high promiscuity as it shows a high activity with the flavonols kaempferol 3-O-glucoside and quercetin 3-O-glucoside. Nevertheless, UDP-xylose is the only accepted sugar donor [50].

Addition of acyl groups to anthocyanins is another common modification of anthocyanins. In crops alone, various acylated anthocyanins were reported in 23 species [81]. Acylation of anthocyanins leads to higher activity, increased stability, and altered polarity which enhances the antioxidant effect and enables the usage as dye in industry [81]. In *A. thaliana*, the majority of the known anthocyanin-related acyltransferases belong to the BAHD superfamily. For *At*3AT1 (*At1g03940*) and *At*3AT2 (*At1G03495*), different acyl donors (p-coumaroyl-CoA, feruloyl-CoA and caffeoyl-CoA) and acyl acceptors (cyanidin 3-glucoside, pelargonidin 3-glucoside, malvidin 3-glucoside, quercetin 3-glucoside, kaempferol 3-glucoside and kaempferol 7-glucoside) are known [51]. *At*5MAT (At3g29590) also has a high promiscuity, as it acylates multiple 3-O-glycosylated anthocyanins, i.e. cyanidin 3,5-diglucoside, cyanidin 3-coumaroylglucoside 5-glucoside, cyanidin 3-O-(xylosyl) 6’’-O-p-coumaroyl glucoside 5-O-glucoside (A3) and delphinidin 3-coumaroylrutinoside 5-glucoside [51]. However, SCPL10, belonging to the serine carboxypeptidase-like gene family, catalyzes a sinapoyl-glucose dependent acylation of anthocyanins in *A. thaliana* [53].

### Transcriptional regulation of anthocyanin biosynthesis

**Fig. 3:**
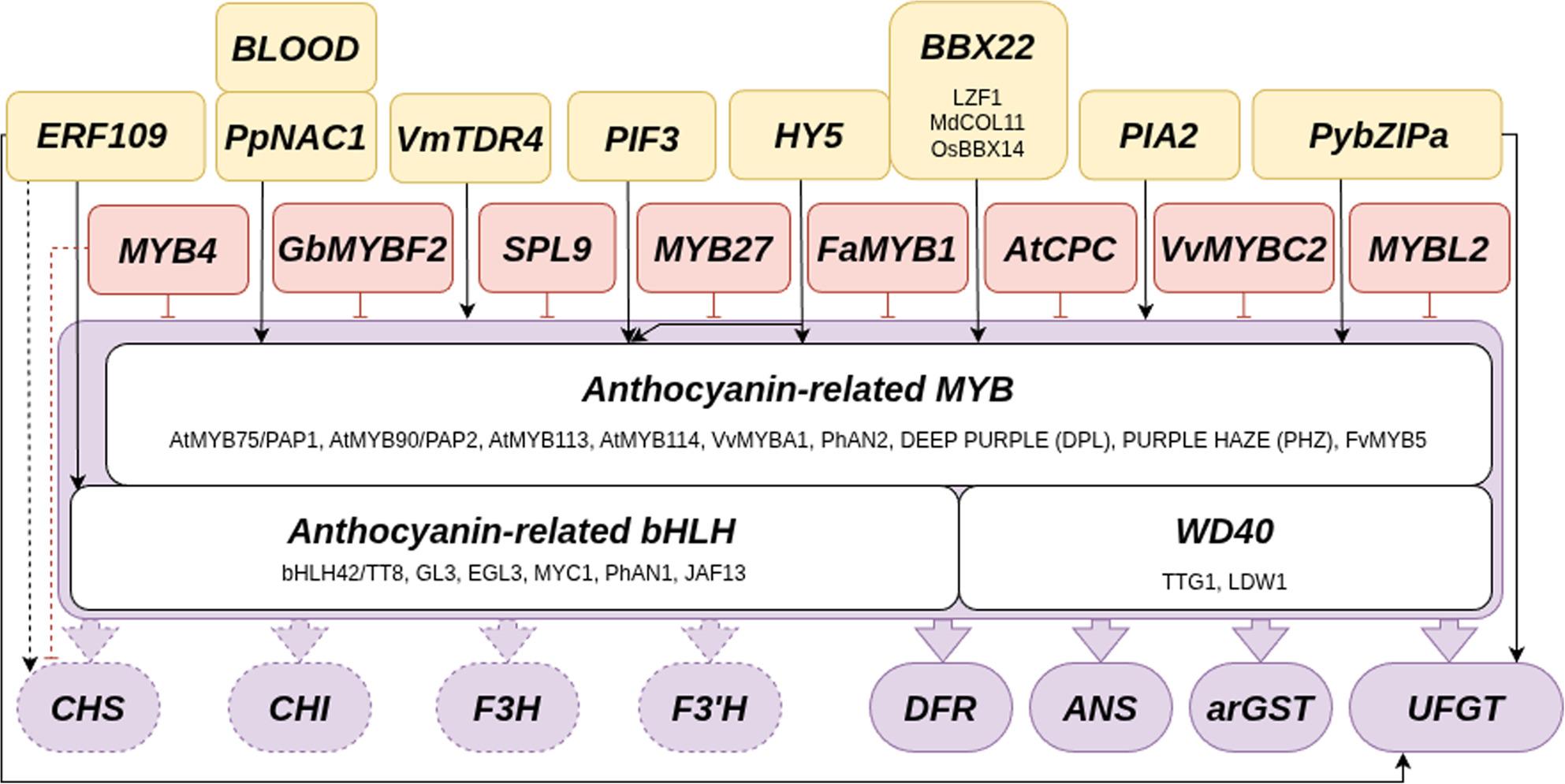
Transcription factors influencing the activity of anthocyanin biosynthesis genes identified in different species. Multiple transcription factors are promoting the anthocyanins biosynthesis through the activation of the anthocyanin biosynthesis regulating MYB and the MBW complex (yellow). Displayed transcription factors are ethylene responsive factor 109 (ERF109), blood, nam - ataf1- cuc2 (NAC1), vaccinium myrtillus tapetum degeneration retardation 4 (VMTDR4), phytochrome interacting factor 3 (PIF3), long hypocotyl 5 (HY5), b-box domain protein 22 (BBX22), phytochrome-interacting ankyrin repeat protein 2 (PIA2), *pyrus pyrifolia* bzipa (pybzipa). Repressors of the anthocyanin biosynthesis (red) are MYB4, Ginkgo biloba MYBF2, squamosa promoter binding protein-like 9 (SPL9), MYB27, *Fragaria × ananassa* MYB1, *Arabidopsis thaliana* caprice (AtCPC), *Vitis vinifera* MYBC2, and MYBL2.. Activations are shown by arrows, dashed arrows indicate involvement in early anthocyanin biosynthesis, and blunt-ended lines represent repression [109, 112–117, 121, 122]. The myeoblastosis - basic helix-loop-helix – WD40 (MYB-bHLH-WD40, MBW) complex consists MYB75/PRODUCTION OF ANTHOCYANIN1 (PAP1), MYB90/PAP2, MYB113, MYB114, *Vitis vinifera* MYBA1, *Petunia hybrida* anthocyanin 2 (*Ph*AN2), deep purple (DPL), purple haze (PHZ) or MYB5 and BHLH42/transparent testa 8 (TT8), glabra 3 (GL3), enhancer of glabra 3 (EGL3), myelocytomatosis 1 (MYC1), *petunia hybrida* anthocyanin 1 (PHAN1) or johnandfranscesca 13 (JAF13) and transparent testa glabra 1 (TTG1) and light-regulated wd1 (LWD1). Displayed structural genes are chalcone synthase (CHS), chalcone isomerase (CHI), flavanone 3-hydroxylase (F3H), flavonoid 3’-hydroxylase (F3’H), dihydroflavonol 4-reductase (DFR), anthocyanidin synthase (ANS), anthocyanin-related glutathione S-transferase (arGST) and UDP-dependent flavonoid-3-O-glycosyltransferase (UFGT). Transcription factors without a species prefix were identified in *Arabidopsis thaliana* or multiple species

Crucial for the metabolic flux control through the flavonoid biosynthesis is the transcriptional regulation of enzyme-encoding genes (Fig. 3). Many MYB transcription factors, members of the largest transcription factor family in plants, play crucial roles in this regulation [16, 82–84]. Genes of the anthocyanin biosynthesis branch are activated by a complex of multiple transcription factors including a MYB, a bHLH, and a WD40 protein leading to the name MBW complex [85, 86]. It is generally assumed that the MYB component is determining the target gene specificity of this complex, because different MYB proteins can be incorporated into this complex leading to variations in the set of target genes [16, 87, 88]. Although the idea of a single transcription factor complex regulating all anthocyanin biosynthetic genes appears straightforward, extensive gene duplications across various taxonomic levels have rendered the regulatory network highly complex. Initially, genes in the flavonoid biosynthesis have been classified as early (*CHS, CHI, F3H*) and late (*DFR, ANS, UFGT*) due to the assumption that the transcriptional control would be distinct. However, discoveries of several transcription factors contributing to the anthocyanin biosynthesis regulation question this system.

### Lineage-specific differences in the MBW complex

Since anthocyanin activating MYBs have been investigated in a large number of plant species, a pattern emerged that implicated different MYB subgroup 6 (SG6) members in the activation of the anthocyanin biosynthesis. They are generally interacting with bHLHs of the IIIf subgroup. Initial studies in *Zea mays* discovered *R* and *B* as two copies of an anthocyanin-regulating bHLH gene [89]. COLORLESS1 (C1) is a MYB transcription factor that was also identified as a crucial factor in the anthocyanin biosynthesis regulation of maize [90]. In *A. thaliana* the MYB75 (PAP1), MYB90 (PAP2), MYB113, and MYB114 are anthocyanin regulators and also very close homologs suggesting an evolutionarily recent radiation [86, 91]. Anthocyanin activating MYBs from monocot species like banana do not efficiently interact with the same bHLHs as MYBs from dicots like *A. thaliana* [92, 93]. There is also an additional component, the WRKY protein TTG2, that can be associated with the MBW complex [94–96]. The current model suggests that *TTG2* is activated by the MBW complex and that the addition of the WRKY component to the MBW complex increases the specificity for the vacuolar transport associated genes *TT12* and *TT13* [88, 96]. *PH3* is a petunia gene with a function similar to the *Arabidopsis TTG2* [95] thus suggesting that the involvement of WRKY is conserved across plant species. Anthocyanin biosynthesis activating WRKYs have also been described in pear and apple, but at least apples MdWRKY40 is reported to be functionally divergent from PH3 and TTG2 [97, 98].

There are also reports that anthocyanin biosynthesis activation is possible without the canonical MBW complex. A recent study in blueberry revealed that proanthocyanidin biosynthesis regulators like TT2/MYB123 (subgroup 5, SG5) can contribute to the anthocyanin regulation [99]. Several studies suggested that MYB5 orthologs are also able to activate the anthocyanin biosynthesis [100–102]. In *Vitis*, *Vv*MYB5a and *Vv*MYB5b can activate specific genes in the anthocyanin biosynthesis with VvMYB5b being able to slightly trigger the accumulation of anthocyanins when heterologously expressed in unpigmented petunia [100]. Additionally, it seems that only *Vv*MYB5a and *Vv*MYB5b, but not the MYB75/PAP1/AN2 ortholog *Vv*MYBA1 are able to activate the *HYDROXYLATION AT FIVE* (*HF2*, *F3’5’H*) expression [100]. It is important to note that *Vitis* also harbors *Vv*MYBA1 and *Vv*MYBA2 as orthologs of *Ph*AN2 which seem to be the dominant activator of most anthocyanin biosynthesis genes [100, 103–105]. Studies in strawberries reported MYB5 as an anthocyanin regulator that is TTG1-independent, but forms a MBW complex with EGL3 (bHLH) and LWD1 (WD40) instead [101]. Reports in other species also associated MYB5 with anthocyanin biosynthesis: PhPH4, also belonging to the MYB5 lineage, regulates vacuolar acidification in petunia [106], and SmMYB5 activates anthocyanin biosynthesis in eggplant [102]. Additionally, *MYB5a/NEGAN* is responsible for the pigmentation pattern in *Mimulus* [107]. While studies in many species identified MYB5 orthologs as anthocyanin biosynthesis regulators *CsMYB5a* and *CsMYB5e* in *Camellia sinensis* activate proanthocyanidin biosynthesis, but not anthocyanin biosynthesis [108] and *A. thaliana MYB5* seems not tightly connected to the anthocyanin biosynthesis. In summary, MYBs of the SG6 are important regulators of the anthocyanin biosynthesis, but some members of the SG5 lineage might also activate specific genes in the anthocyanin biosynthesis. This could represent a mechanism for fine-tuned activation of specific branches within the complex anthocyanin metabolism, but further systematic investigations are required.

### Additional transcription factors influence anthocyanin biosynthesis

A number of other transcription factors have been implicated in the regulation of anthocyanin biosynthesis genes including ethylene responsive factor 109 (ERF109), NAM/ATAF/CUC2 (NAC), MADS-box, basic leucine zipper (bZIP), B-box domain protein 22 (BBX), and phytochrome interacting factor 3 (PIF3). Many of these transcription factors activate genes encoding MBW components rather than the actual anthocyanin biosynthesis genes. In *Malus domestica*, ERF109 activates the anthocyanin biosynthesis in early stages of apple coloration by binding promoters of *MdCHS*, *MdUFGT*, and *MdbHLH3* [109]. Another study in red-skinned pear reported the interaction of *Py*ERF with the anthocyanin regulators *Py*MYB114 and *Py*bHLH3 to co-regulate the anthocyanin biosynthesis [110]. NAC proteins have been identified as activators of the anthocyanin biosynthesis in *Arabidopsis thaliana* and *Prunus persica* [111, 112]. NACs probably trigger anthocyanin pigmentation through up-regulation of the important anthocyanin biosynthesis activating MYB as this was observed for a BLOOD/PpNAC1 heterodimer in the coloration of blood-fleshed peach during fruit ripening [112]. Since the expression pattern of the SQUAMOSA-class MADS-box gene tapetum degeneration retardation 4 (TDR4) is tightly correlated with the anthocyanin pigmentation in bilberry fruits (*Vaccinium myrtillus*), it was postulated that this transcription factor directly or indirectly controls the anthocyanin biosynthesis genes [113]. In *A. thaliana*, the bZIP protein long hypocotyl 5 (HY5) binds to the promoter of *MYB75/PAP1* and triggers the anthocyanin biosynthesis through up-regulation of this MYB gene [114]. This result is supported by a study in *Malus domestica* that identified MdHY5 as an activator of the anthocyanin regulator *Md*MYB10 [115]. Another bZIP protein, PybZIPa, was reported as an activator of the anthocyanin activating MYB and UFGT, but not orthologous to HY5 [116]. The *A. thaliana* BBX protein BBX22/LZF1 was identified as an activator of the anthocyanin-regulating *MYB75/PAP1* [117]. HY5 was discovered as a factor contributing to the expression of BBX22/LZF1 [117]. The apple BBX protein *Md*COL11, ortholog of *At*BBX22, is interacting with *Md*HY5 to activate the anthocyanin regulator *Md*MYBA [118]. A study in rice identified that *Os*BBX14 and *Os*HY5 interact to activate the anthocyanin regulating MYB and bHLH gene [119]. A study in red pear revealed that *Pp*BBX16 interacts with *Pp*HY5 to activate the expression of anthocyanin biosynthesis genes [120]. In summary, this suggests that HY5 usually acts in a complex with a BBX protein. In *A. thaliana*, *PIF3* is another light-responsive transcription factor that can activate the anthocyanin biosynthesis, but requires the simultaneous binding of HY5 [121]. Another positive regulator of the anthocyanin biosynthesis in *A. thaliana*, especially associated with up-regulation of the *UFGT*, is the phytochrome-interacting ankyrin repeat protein 2 (PIA2) [122]. MYB112 was reported to trigger anthocyanin biosynthesis and block flavonol biosynthesis under salt and high light stress conditions, by activating the anthocyanin activator PAP1 and repressing the flavonol activators MYB12/MYB111 [123]. Not all transcription factors of the anthocyanin biosynthesis are activators. A comprehensive review summarized the role of various factors that repress the expression of anthocyanin biosynthesis genes [84]. Examples for characterized MYB repressors connected to the anthocyanin biosynthesis are *Ph*MYB27 in petunia [124], FaMYB1 in strawberry [125], CAPRICE (CPC) in *Arabidopsis* [126], SQUAMOSA PROMOTER BINDING PROTEIN-LIKE 9 (SPL9) group in *Arabidopsis* [127], MYBL2 in Arabidopsis [128], MYBC2-L1 and MYBC2-L3 in grapevine [129], MYBF2 in ginkgo [130], and *Ma*MYB4 in banana [131]. Many studies about regulators of the anthocyanin biosynthesis have observed this role only in a single plant species. It was previously observed that changes in the transcriptional regulation explain a majority of evolutionary anthocyanin pigmentation shifts at the species and genus level [16]. This suggests a high flexibility of the transcriptional control of the anthocyanin biosynthesis during evolution. Therefore, it is feasible that at least some of the large number of reported regulators are species- or lineage-specific features of the anthocyanin biosynthesis. There is a need for systematic studies that explore these transcription factors across a wide range of different plant species.

### Regulatory RNAs in the anthocyanin biosynthesis

Activity of the anthocyanin biosynthesis is also controlled by regulatory RNAs. There are microRNAs (miRNAs) that suppress target genes, small RNAs that suppress their target gene, and long non-coding RNAs (lncRNAs) that counteract the miRNAs by target mimicry, i.e., sequestering miRNAs without cleavage of the lncRNAs [132–135]. Expression patterns of certain lncRNAs were associated with the accumulation of anthocyanins, but evidence for the molecular mechanism is missing [136]. A small RNA originating from an inverted duplication at the SULF locus results in suppression of the 4’CGT involved in aurone biosynthesis, which causes an increased synthesis of anthocyanins [135]. Anthocyanin accumulation in *Arabidopsis* is increased by miR156 that targets anthocyanin repressors of the SPL9 group [127]. The authors demonstrated that SPL9 captures PAP1 proteins and prevents the formation of the MBW complex that is required for the activation of anthocyanin biosynthesis genes. A similar system comprising miR156a and SPL9 as negative regulators of the anthocyanin biosynthesis was reported as the mechanism explaining the coloring of the peel in red pear [137]. Two long non-coding natural antisense transcripts (lncNATs) have been reported as repressors of the anthocyanin biosynthesis activators DcMYB6 and DcMYB7 [138]. A regulatory system identified in *Malus spectabilis* under nitrogen starvation comprises miR858 as a repressor of MYB62-like, which in turn represses the anthocyanin biosynthesis, thus high expression of miR858 leads to anthocyanin accumulation [134]. This study also identified eTM858-1 and eTM858-2, two target mimics of miR858, which reduce the activity of miR858 on MYB62-like thus leading to a repression of the anthocyanin biosynthesis [134]. In sea buckthorn, LNC1 and LNC2 (TCONS_00694050 and TCONS_00438839) were reported as endogenous target mimics of miR156a and miR828a, respectively [133]. While miR156a targets the anthocyanin biosynthesis repressor SPL9, miR828a targets the anthocyanin biosynthesis activator MYB114 [133]. Consequently, a high abundance of LNC1 leads to reduced anthocyanin accumulation in the fruit, while higher abundance of LNC2 results in a higher anthocyanin accumulation [133]. In apple, MdLNC499 activates the expression of *MdERF109*, which encodes a transcriptional activator of the anthocyanin biosynthesis genes *CHS*, *bHLH3*, and *UFGT* [109].

A large proportion of knowledge about the anthocyanins biosynthesis originates from research on *A. thaliana*, which limits the potential for discoveries of regulatory mechanisms present in this species. The anthocyanin biosynthesis is generally considered one of the best studied pathways in plants. It is widely believed that this pathway including its regulation is well conserved across plants, which might be reinforced by numerous recent studies only focusing on the well characterized genes of the core anthocyanidin biosynthesis in new plant species. In the light of recent discoveries summarized above, it appears highly likely that there are lineage-specific features of the anthocyanin biosynthesis that could be discovered through comparative investigations.

### Transport of anthocyanins

**Fig. 4:**
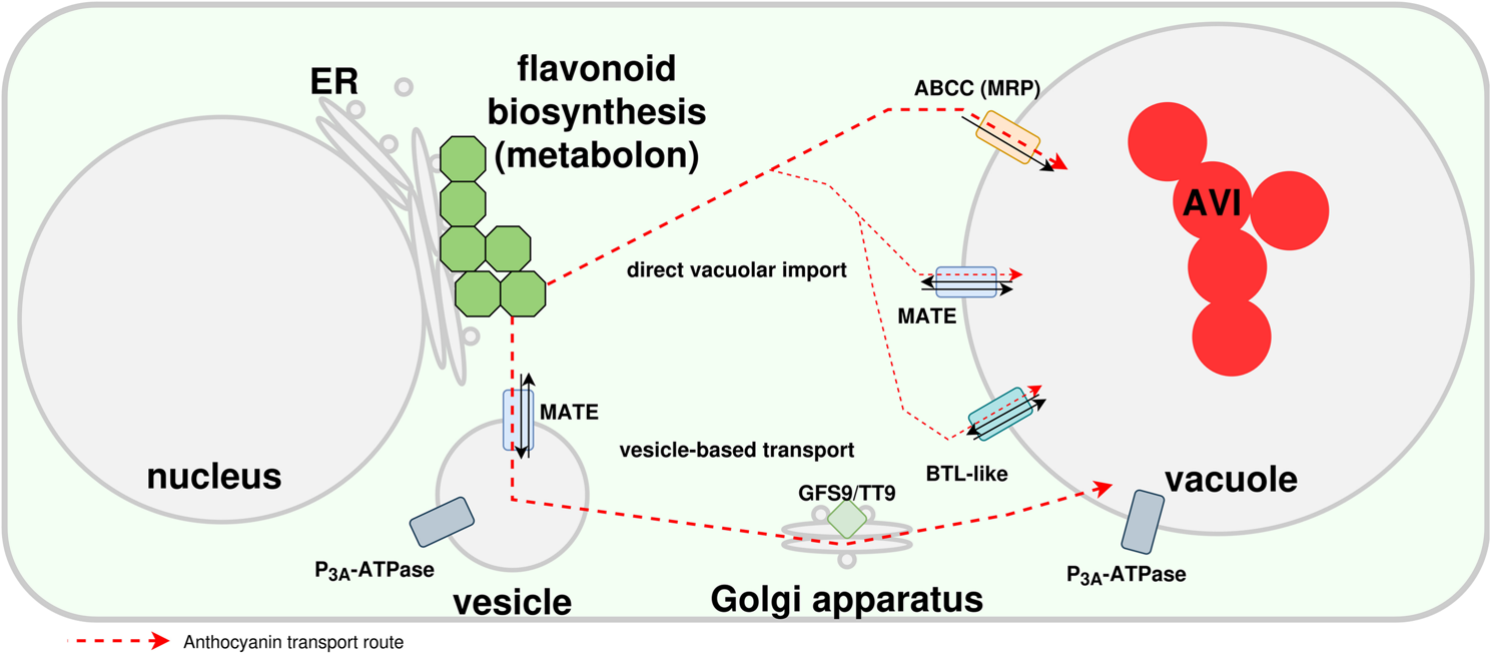
Simplified illustration of proposed anthocyanin transport routes from the endoplasmic reticulum (ER) to the central vacuole. Figure is based on a previous version published in Pucker & Selmar [139]. Shown are ABCC (ATP-binding cassette (ABC) subfamily C), MATE (Multidrug And Toxin Extrusion transporter), BTL-like (bilitranslocase-like), GFS9/TT9 (Green Fluorescent Seed 9/Transparent Testa 9), and AVI (anthocyanin vacuolar inclusion). Green hexagons represent the anthocyanin biosynthesis enzymes at the ER

Anthocyanins are produced at the endoplasmatic side of the ER and require transportation into the central vacuole for long term storage [139] (Fig. 4). Details regarding the intracellular transport of anthocyanins have eluded researchers as results of previous studies seem to contradict each other. Different ABCC proteins have been implicated in the transport of anthocyanins across a membrane [140–142]. However, it is not clear whether anthocyanins are imported into the ER and transferred to the vacuole or transported through the cytoplasm and then imported into the central vacuole [139]. The mechanism could differ between plant species or both routes could contribute to the anthocyanin transport. The discovery of TT9, a vesicle associated protein, as a crucial factor for proanthocyanidin pigmentation in *A. thaliana* [143] could suggest that anthocyanin transport is also occurring through vesicles. MATE (TT12) transporters have also been proposed as potential anthocyanin transporters [144]. They would require a proton gradient that could be maintained by AHA10/TT13 [145]. However, it was also suggested that MATEs are not transporting anthocyanins, but proanthocyanidin precursors [146]. Previously, it was postulated that TT19, a glutathione S-transferase (GST), serves as ‘ligandin’, an anthocyanin protection protein, during the transport through the cytoplasm [147–149]. However, a recent study demonstrated an enzymatic function of this anthocyanin-related GST (arGST) in the synthesis of cyanidin [33]. While this finding does not rule out an arGST function in the transport of anthocyanins, it provides an alternative explanation for previous observations that led to the postulation of the arGST function in the anthocyanin transport. Understanding the transport of anthocyanins is crucial as some modification reactions take place in the central vacuole, i.e., after the successful translocation [150]. Anthocyanin localization within the cell can influence the phenotypic appearance of the plant. Clusters of anthocyanins can be dispersed rapidly, altering the color of the plant organ [151]. Changes in the vacuolar acidity can quickly change the appearance of anthocyanins as these are depending on the pH [152]. While there is substantial research on the intracellular transport of anthocyanins, not much is known about the transport of anthocyanins between plant cells or even different organs [139]. Since all plant cells should be able to produce anthocyanins based on phenylalanine, transport between cells might not occur at a relevant level.

### Cell-specific accumulation of anthocyanins and pigmentation patterns

Pigmentation patterns mostly serve the attraction of pollinators by boosting the visibility of the flower to increase the number of visitation events. Such pigment cues are widespread across angiosperms and represent a general strategy to optimize pollinator foraging efficiency [153, 154]. This can be achieved through high contrast patterns or special flower patterns [153–155]. These floral patterns are usually colored in a shade or color contrasting the rest of the flower and provide information for the pollinator on the location and orientation for the landing on the flower [156]. From there, spatially pigmented nectar guides can lead the way to the nectaries thus substantially increasing pollination success [155, 157]. Anthocyanin pigmentation patterns can usually be attributed to the spatial expression of general flavonoid and particularly anthocyanin biosynthesis related genes. Anthocyanin biosynthesis genes are regulated by an ensemble of transcription factors that are active in very specific flower parts and tissues [155, 158, 159]. The anthocyanin biosynthesis promoting MYB is the most specific transcription factor in most plant species [16]. Different tissue types of the flower petal can contribute to specific spatial pigmentation of the tissue or the adjacent cells. This can be seen in *Antirrhinum* in the phenotype Venosa where the transcription factor VENOSA from the vascular tissues leads to red coloration of the adjacent adaxial epidermis [160]. In *Petunia hybrida*, the MADS-box gene *DEFICIENS* was reported as a cell layer-specific transcription factor in flowers that influences the pigmentation pattern through control of the expression of the major anthocyanin biosynthesis regulating MYB *ANTHOCYANIN2* [161]. In *Gossypium barbadense,* a MYB transcription factor is essential for the formation of one large spot at the base of the petal which attracts pollinators to the flowers more efficiently [162]. The petal spot formation in *Gorteria diffusa* is also controlled by an anthocyanin MYB that triggers the malonylation of anthocyanins specifically in this petal region [163].

**Fig. 5:**
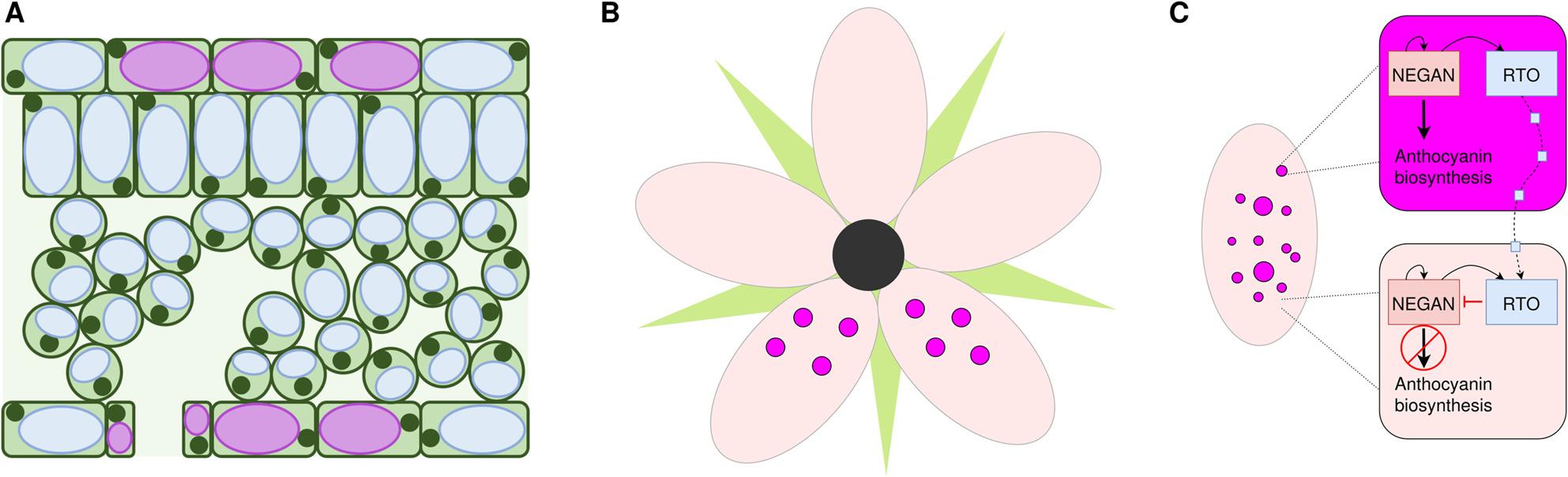
Anthocyanin pigmentation patterns in petals. This cross-section shows that individual cells in specific layers can be colored by anthocyanins, while adjacent cells do not accumulate anthocyanins (**A**). Patterns on flowers emerge due to anthocyanin accumulation in restricted areas like a bull’s eye in the center of a flower or intensely pigmented spots on the petals (**B**). The formation of pigmented spots can be explained by an activator-repressor model as proposed by Ding *et al* [159] (**C**)

Several plant species display intensely pigmented spots on their petals that appear to be randomly distributed. For example, in *Mimulus lewisii*,* Mimulus guttatus*, and *Digitalis purpurea* the lower petal contains an area with multiple spots contrasting the background [159, 164, 165]. This area is centered on the petal and forms nectar guides in *Mimulus* [159] and a putative landing site in *Digitalis* [165]. In *Mimulus*, it was shown that the basis of spot formation is an activator-repressor-relationship of two transcription factors (Fig. 5), which is conforming with a reaction-diffusion model proposed by Turing and Gierer & Meinhardt [159, 166, 167]. This model assumes a relationship between an activator and a repressor, in which the activator (in this case the R2R3-MYB *NEGAN*) enhances its own expression and, in this case, upregulates the anthocyanin biosynthesis genes [159]. Through diffusion of the activator into adjacent cells, an anthocyanin-pigmented spot develops [159]. At the same time the activator upregulates a repressor gene (here: R3-MYB MlRTO) which competes with the activator for binding with the bHLH and WD40 partners [159]. Thereby it reduces the functionality of the activator and therefore the expression of anthocyanin biosynthesis genes [159]. Both regulators diffuse over cell boundaries. The repressor diffuses faster and represses the anthocyanin biosynthesis gene activity in cells adjacent to the cells where the activator is active [159]. The pigmented spot is confined to the specific boundaries being set by the diffusion- and degradation-properties of both transcription factors. White halos surrounding pigmented spots on otherwise differently pigmented backgrounds can be explained by the repressor activity in the spot-adjacent cells [155, 159]. These examples of spatially restricted pigmentation illustrate the diversity of floral color patterns, which can arise through different molecular mechanisms. Another striking case presents itself in the *Petunia hybrida* ‘Red Star’ variety. Flowers of this variety display a white star-like pattern on a red petal background [168]. It was shown that spatial post transcriptional gene silencing of the *PhCHS A* mRNA is responsible for the lack of pigmentation around the center vein of the petals [168]. The resulting lack of CHS A enzymes in this tissue leads to the disruption of the early flavonoid pathway and a subsequent block of anthocyanin production.

Depending on their localization in certain cell layers (spongy palisade mesophyll, upper epidermis, lower epidermis), the same anthocyanins can lead to different colors of plant structures as recently observed in leaves of *Tipularia discolor* [25]. A large study investigating hundreds of plant species reported that mesophyll is the most frequent location of anthocyanins and only 24% of the studied species displayed anthocyanins in the epidermis [169]. The huge diversity of anthocyanin accumulation patterns reported is strong support for a broad range of different anthocyanin functions [169, 170]. In contrast, anthocyanins have been reported to accumulate in the epidermis of *A. thaliana* plants under nitrogen starvation [171]. A study on various crucifers also reported high anthocyanin concentration in the epidermis with spread into the mesophyll occurring only at very high anthocyanin concentrations [172]. Understanding the localization of anthocyanins inside plants is a remaining challenge that can help to understand their physiological function and evolutionary relevance.

### Ecological functions of anthocyanins

Functions of anthocyanins might differ depending on developmental state, plant organ, or environmental conditions. For example, anthocyanins in leaves primarily provide protection against high light intensities and other stress factors [1, 173]. In flowers and fruits, anthocyanins are mainly involved in pollinator and seed disperser attraction, but also contribute to protection against environmental stresses [174–179]. Understanding the ecological functions of anthocyanins is crucial when generating hypotheses about evolutionary trajectories that lead to the anthocyanin biosynthesis as we see it today. For example, the loss of anthocyanins may coincide with changes in the pollination system or in seed dispersal syndrome.

### Protective functions of anthocyanins in photosynthetically active plant organs

Anthocyanins protect green plant parts from excessive light intensities and oxidative stress. While the leaves of many plants turn red in response to high light intensities [180, 181], other species show constant red leaf pigmentation [182–184]. The red color of anthocyanins is due to a strong absorbance of green and blue-green light which could damage cell components [185–187]. Anthocyanins can act as sunscreen shielding the leaf tissue from excess light that would otherwise be captured by chlorophyll b [182, 188, 189]. The maximum photosynthesis of leaves with anthocyanin pigmentation was increased compared to green leaves and photoinhibition was reduced [182, 190]. The red undersurface of understory plants or floating leaves of aquatic plants is probably not backscattering red light as initially hypothesized, but helps the plants to cope with high-intensity sun flecks [182, 191]. In addition to protection against excess light, anthocyanins have also been reported as antioxidants that quench reactive oxygen species (ROS) that could otherwise damage cell structures [192, 193]. While ROS are mainly produced in chloroplasts, anthocyanins are transported into and stored in the central vacuole [139]. This brings up the question how anthocyanins can counteract ROS without physical proximity. Photooxidative stress leads to the inactivation of the ascorbate peroxidase, an enzyme that normally degrades H_2_O_2_ [193, 194]. H_2_O_2_ is the ROS type that temporarily accumulates in plastids under photooxidative conditions and can move into the vacuole either through passive diffusion or through proteins located in the tonoplast [195–197]. The anthocyanin-filled vacuole would become a H_2_O_2_ sink which can explain the ROS scavenging through anthocyanins accumulated in the vacuole probably catalyzed by peroxidases [193, 198, 199]. This hypothesis aligned with the observation that ROS quenching by anthocyanins is substantially weaker in species like *Rosa sp.* and *R. communis* where anthocyanins are not located in the same cells as the plastid accumulating H_2_O_2_ [193]. A systematic study revealed that the photoprotective function of anthocyanins is especially important at low temperatures and high light intensities, when other photoprotection mechanisms are less effective [200]. Anthocyanin levels might also be regulated in response to ROS to ensure that excess ROS is scavenged [201]. The photoprotective role of anthocyanins is not restricted to leaves, but also extends to photosynthetically active stems, flowers, and fruits [178, 202]. A protective function of anthocyanins could explain their presence in young leaves, seedlings, or developing fruits [203–206]. These emerging structures require a protective anthocyanin pigmentation, because light capture ability develops before CO_2_ assimilation capacity thus protection against photoinhibition is needed [207, 208]. The *de novo* synthesis of anthocyanins in senescent leaves during autumn did appear less obvious and turned into an intensely studied field with many hypotheses arising to explain this phenomenon [188, 209–212]. Anthocyanins can delay senescence and ensure functionality of leaves which improves the resorption of nitrogen and phosphorus in autumn [188, 213, 214]. Anthocyanin formation during autumn leading to red leaves is more pronounced in species that grow in areas with shorter vegetation periods and are more often experiencing cold snaps [212]. In light of the crucial role of anthocyanins in photoprotection, it has been proposed that this physiological function in photosynthetic tissues evolved prior to their role as visual cues for pollinators in floral tissues [182].

### Importance of anthocyanins in drought and salt stress response

Plants under drought stress caused by limited water availability or increased salt concentrations were often observed to turn red through the accumulation of anthocyanins. Studies exploring the transcriptomic or metabolomic changes report a generally increased activity of the flavonoid biosynthesis without specific explanations how the anthocyanin biosynthesis is increased [215–218]. Therefore, it is plausible that ROS scavenging is performed by anthocyanins and other flavonoids, i.e., the enrichment of anthocyanins is the consequence of generally increased activity of the flavonoid biosynthesis. A specific function of anthocyanins might be capturing excess light as photosynthesis cannot take place in the absence of water. In *Arabidopsis*, the two glycosyltransferases UGT79B2 and UGT79B3 were identified as important targets for activation of the anthocyanin biosynthesis under drought and salt stress conditions, but also in response to cold stress [219]. The importance of flavonols in response to salt stress was investigated based on flavonol deficient mutants that were more adversely affected than the wild type [220]. Given that the flavonol and anthocyanin biosynthesis branches are competing for shared substrates [221], this observation suggests that generally antioxidants, but not specifically anthocyanins, are needed under these stress conditions. An investigation of carrot cell cultures under salt stress discovered the importance of MATE in the increased anthocyanin accumulation [222], which could indicate that a generally increased transport across membranes to counteract the osmotic challenges increases anthocyanin transport as a side effect. Anthocyanins account for less than 1% of the osmotic potential in a plant cell which makes a role as compensating salts unlikely [211, 223]. The sparsity of data about anthocyanin regulation in response to drought and salt stress suggests that their role in this context is rather minor and thus more challenging to resolve. This suggests that drought and salt stress response were not among the factors that contributed to the evolution of the anthocyanin biosynthesis. Although genetically engineered crops with increased anthocyanin content have been shown to have an enhanced resistance to salt and drought stress, further knowledge about regulation mechanisms in this process is required to enable effective applications [224]. It has been suggested that betalains, which replace anthocyanins in many Caryophyllales, might play a more important role in salt stress tolerance [225].

### Cold stress response

When exposed to low temperatures, many plants turn red due to accumulation of anthocyanins. This color change is caused by transcriptional up-regulation of the genes involved in the anthocyanin biosynthesis [203]. Some evergreen species that maintain their leaves over the winter turn completely red due to intense anthocyanin accumulation [226, 227]. Initially, it was postulated that anthocyanins could potentially turn light into heat thus increasing the plant temperature [228], but presence in tropical plants makes this hypothesized function of foliar anthocyanins unlikely [182, 214, 229]. Following the observation that light at low temperatures is particularly harmful for plants, it appears more likely that anthocyanins in evergreens protect against photoinhibition during winter [182, 223, 226, 227]. While a likely anthocyanin function of anthocyanins under cold stress is protection [219, 230] and potentially avoiding a sugar excess, the role of differently modified anthocyanins in cold response remains an open question.

### Anthocyanin accumulation as sign of nutritional imbalance

An imbalance in the availability of sugar, nitrogen, phosphorus, and other nutrients can trigger the accumulation of foliar anthocyanins [231]. Magnesium, sulfur, boron, copper, and sometimes potassium deficiencies can also result in the blushing of plants [231–237], but results for some nutrients are restricted to individual species and limit general conclusions. While sugar and nitrogen are well investigated, little is known about the roles of most other elements. A positive impact of anthocyanins on performance under phosphorus starvation is suggested by an experiment with wheat seedlings [238]. ABI5 might be the interconnection of the anthocyanin boosting ABA signal, light signals, and the phosphor starvation signal in *Arabidopsis* [239]. Results regarding the impact of sulfur starvation on the anthocyanin biosynthesis are inconsistent and might indicate species-specific differences, which have recently been reviewed by Jezek *et al*. [231].

**Fig. 6:**
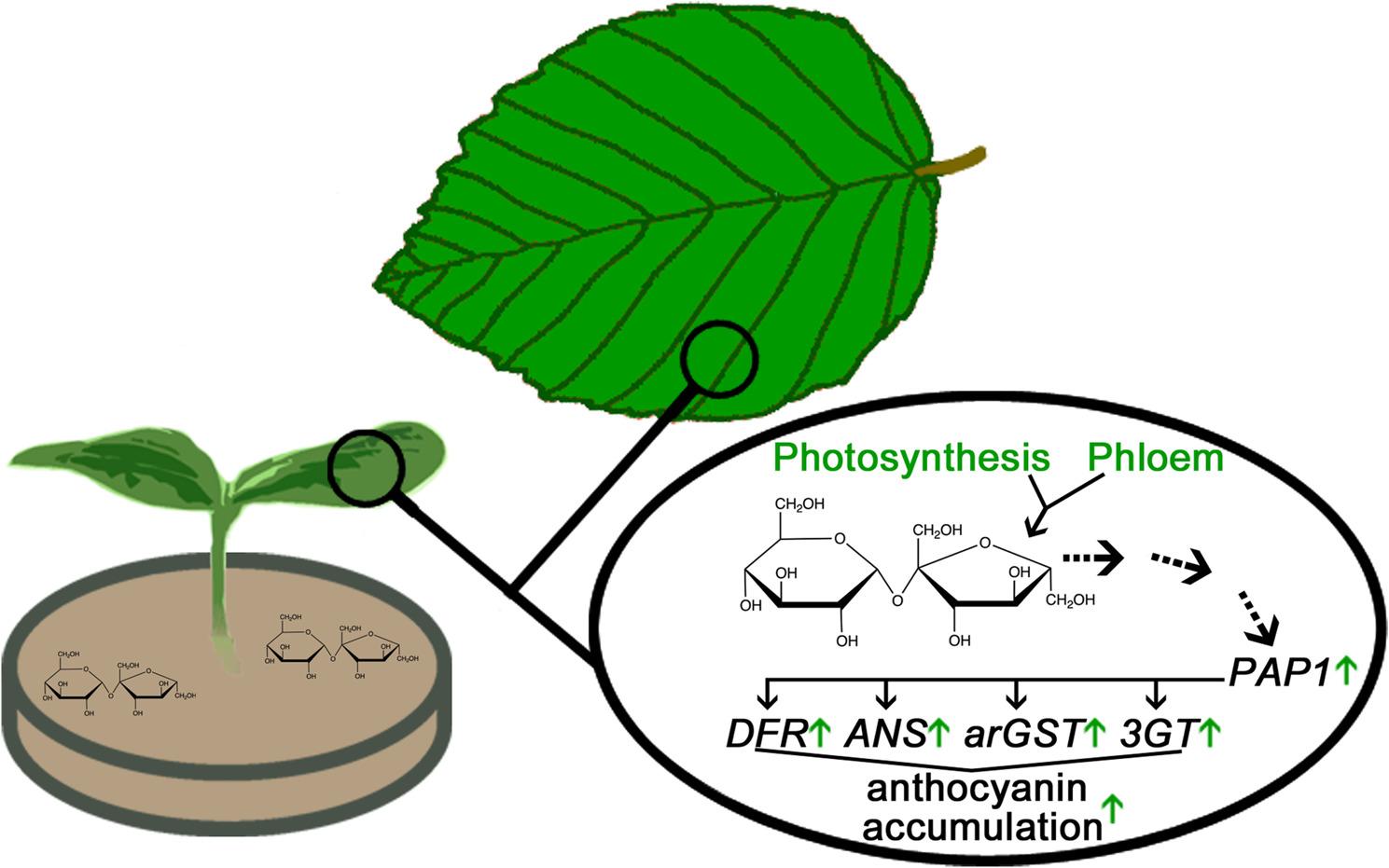
Influence of sucrose on anthocyanin accumulation. Sucrose can either be taken up from the environment, e.g. via sucrose-containing medium (left), or produced through photosynthesis (top). High sucrose concentrations activate the transcription factor PRODUCTION OF ANTHOCYANIN1 (PAP1), which in turn induces general anthocyanin biosynthesis genes such as dihydroflavonol 4-reductase (DFR), anthocyanidin synthase (ANS), anthocyanin-related glutathione S-transferase (arGST), and anthocyanidin-3-O-glucosyltransferase (3GT), resulting in increased anthocyanin accumulation. The exact molecular regulatory mechanism leading to PAP1 activation through sucrose remains unclear

Sucrose and to a lower extent maltose can induce the anthocyanin biosynthesis when added to the growth media of *Arabidopsis* seedlings on agar plates (Fig. 6) [240, 241]. Signals indicating the increased sugar availability or the sugar availability itself could lead to anthocyanin accumulation [242, 243]. There is no evidence for an osmotic effect of sucrose to explain this observation [241]. Instead, it appears that multiple signaling pathways are integrated, ultimately resulting in an activation of the anthocyanin biosynthesis. Previous studies investigated the impact of sucrose on the general flavonoid biosynthesis genes and the anthocyanin biosynthesis genes *DFR*, *ANS*, and *UF3GT* [241, 244, 245]. Additional investigations of the recently reported arGST [33] and various anthocyanin transporters [139] would be important in the future and might help to understand the whole picture. While studies often report the transcriptional up-regulation of individual structural genes in the anthocyanin biosynthesis, it seems more plausible that this is the consequence of transcription factor activation. Given the possibility for extensive decoration with sugars, anthocyanins could represent sugar sinks that would delay the onset of a sugar-promoted leaf senescence by preventing excessive sugar levels [246, 247]. This aligns with the finding that anthocyanin accumulation during high light acclimation appears to depend mostly on the increased cellular sugar content [248] and the importance of arogenate-derived phenylalanine levels for anthocyanin accumulation [249]. Although stress-induced anthocyanins do not degrade immediately after the stress conditions are lifted [250], it is possible that individual sugar moieties might be released. However, based on data from *Elatostema rugosum* [251] and *Zea mays* [252] the amount of carbon stored in anthocyanins was reported as insufficient to account for all accumulating sugar [253]. A more comprehensive database could help to unravel the predominant function of anthocyanins and potential differences between plant species. SUC1 was proposed as a potential integrator of light, sugar, and ethylene signals in the control of the anthocyanin biosynthesis [254, 255] with ethylene having a negative influence on anthocyanin formation [255, 256]. However, a more recent study suggested an intracellular sucrose detection following a SUC1-mediated import as part of the sucrose-induced anthocyanin formation [257]. *PAP1* expression is up-regulated in response to sucrose treatments (Fig. 6), while other sugars do not have the same effect [240, 258, 259]. The circadian rhythms of the *PAP1* expression and the close connection of its expression pattern to intracellular sugar levels suggest that *PAP1* expression might be controlled in vivo by sugar levels [241, 260]. PAP1 specifically up-regulates structural genes of the anthocyanin biosynthesis [258] thus other transcription factors must be involved in activating upstream genes [241]. In contrast to the *PAP1* activation, the expression of its partner *TT8* was reported to be repressed in the presence of sucrose through a signaling pathway comprising YDA-EIN3/EIL1 [256]. MYB30 was recently reported as an inhibitor of MYB75/PAP1 under low sucrose conditions [261]. The postulated regulatory model involves the ubiquitin E3 ligase *RHA2b* as a MYB75 target gene which triggers the degradation of MYB30 under high sucrose levels resulting in higher MYB5 activity and anthocyanin formation [261]. SnRK1 is activated by carbon- and energy-depleting stress and dissociates the MBW complex thus preventing the carbon-intensive biosynthesis of anthocyanins [262]. The MYB75 protein is degraded and TTG1 is exported from the nucleus [262]. An inhibition of the flavonol biosynthesis in *Arabidopsis* seedlings was observed in the presence of high sucrose levels [241], which aligns with the competition mitigation through almost mutually exclusive gene expression of the first committed genes in the anthocyanin and flavonol biosynthesis, respectively [221].

Anthocyanins increase the survival rate under nitrogen starvation [263]. On the molecular level, the decoration patterns of anthocyanins, i.e. the addition of sugar moieties and other functional groups, are altered under nitrogen deficiency [171, 172, 264]. This could be caused through differences in the activation of structural genes through stress-specific transcription factors. MYB90/PAP2 appears as the dominant anthocyanin activator under nitrogen starvation [265, 266], while otherwise MYB75/PAP1 is considered the dominant anthocyanin activator [86, 240]. Translocation of nitrogen into younger tissues and parallel breakdown of chlorophyll causes an initial reddening of blades, veins, and petioles of old leaves [267]. The pigmentation patterns caused by different nutritional imbalances can be distinct: while nitrogen starvation leads to anthocyanin accumulation along the veins, a phosphorus limitation would trigger a reddening between leaf veins [267, 268]. Such differences in blush patterns representative for certain stress conditions might have the potential to be developed into biomarkers [231, 269]. Of particular economic importance are nutritional imbalances influencing the accumulation of anthocyanins in fruits which can alter the commercial value depending on consumer preferences [270].

### Pollinator attraction

Anthocyanins are well known for their contribution to flower colors with a wide range of colors including orange, red, magenta, purple, blue, and black [5, 271]. Flower color is a central visual signal for communication between plants and pollinators that should make the flower stand out from the rest of the plant [272]. Evolutionary changes in flower colors, e.g., blue to red, are often associated with pollinator changes as different pollinator groups have different color perception and resulting preferences [273–275]. Many pollinators can generalize their foraging to an array of different flower colors [276]. A complete loss of anthocyanin pigmentation within a plant species or genus has been frequently observed [16] and might suggest a transition from animal to wind pollination or from color-attracted pollinators to nocturnal insects [277, 278]. However, differences in flower color are not always associated with differences in pollinator attraction [279, 280]. Some communication between plant and pollinator is not visible to humans with the naked eye. For example, flavonol patterns in the UV range can be perceived by animals with a compatible vision, but not by humans [281–284]. In rare cases, the flower color can change following a visitation or pollination event to redirect pollinators to the rewarding flowers [174–177]. The ‘flag hypothesis’ states that old flowers are retained for long-distance attraction of pollinators to the plant [174, 176]. However, flower color change can also be independent of a visitation/pollination event as observed for *Fuchsia excorticata* [285], *Pulmonaria collina* [286], and *Victoria cruziana* [287]. Pollinator attraction might be a derived function of anthocyanins. This is further supported by the superior contrast provided to pollinators by aurones and carotenoids [288]. The widespread presence of anthocyanins in leaves and other green structures suggests that their role in pollinator attraction may be a secondary function that emerged later in evolution [289].

### Seed disperser attraction

Many ripe fruits and berries like apples, pears, blackberries, blueberries, and grapes can be intensely pigmented by anthocyanins [290–293]. The fruit coloration can be a signal of ripe fruits and attract primarily frugivorous birds but also mammals by providing strong contrast to the background [228, 294, 295]. This attraction of birds seems to be important for the seed dispersal [228, 296]. Additional fruit flags that support the attraction of seed dispersers have been described [228]. The accumulation of anthocyanins in fruits like pear is induced by light and involves characteristic WRKY transcription factors including *Py*WRKY26 [98] and *Pp*WRKY44 [297]. HY5 is a central light-responsive regulator that activates the anthocyanin biosynthesis promoting MYB genes and genes of the anthocyanin biosynthesis directly [114]. It has been reported that the proteins BBX16 and BBX18 are interacting with HY5 in this light-dependent activation of the anthocyanin biosynthesis in pear [120, 298]. To the best of our knowledge, there are no conclusive experiments showing the evolutionary benefit of light-induced formation of anthocyanins in fruits yet. It appears plausible that this is a derived property from the regulation of the anthocyanins in leaves, where light responsive formation of protective anthocyanins has obvious advantages, and could suggest that anthocyanins have also a protective function in fruits.

### Herbivore repellence and pathogen resistances

**Fig. 7:**
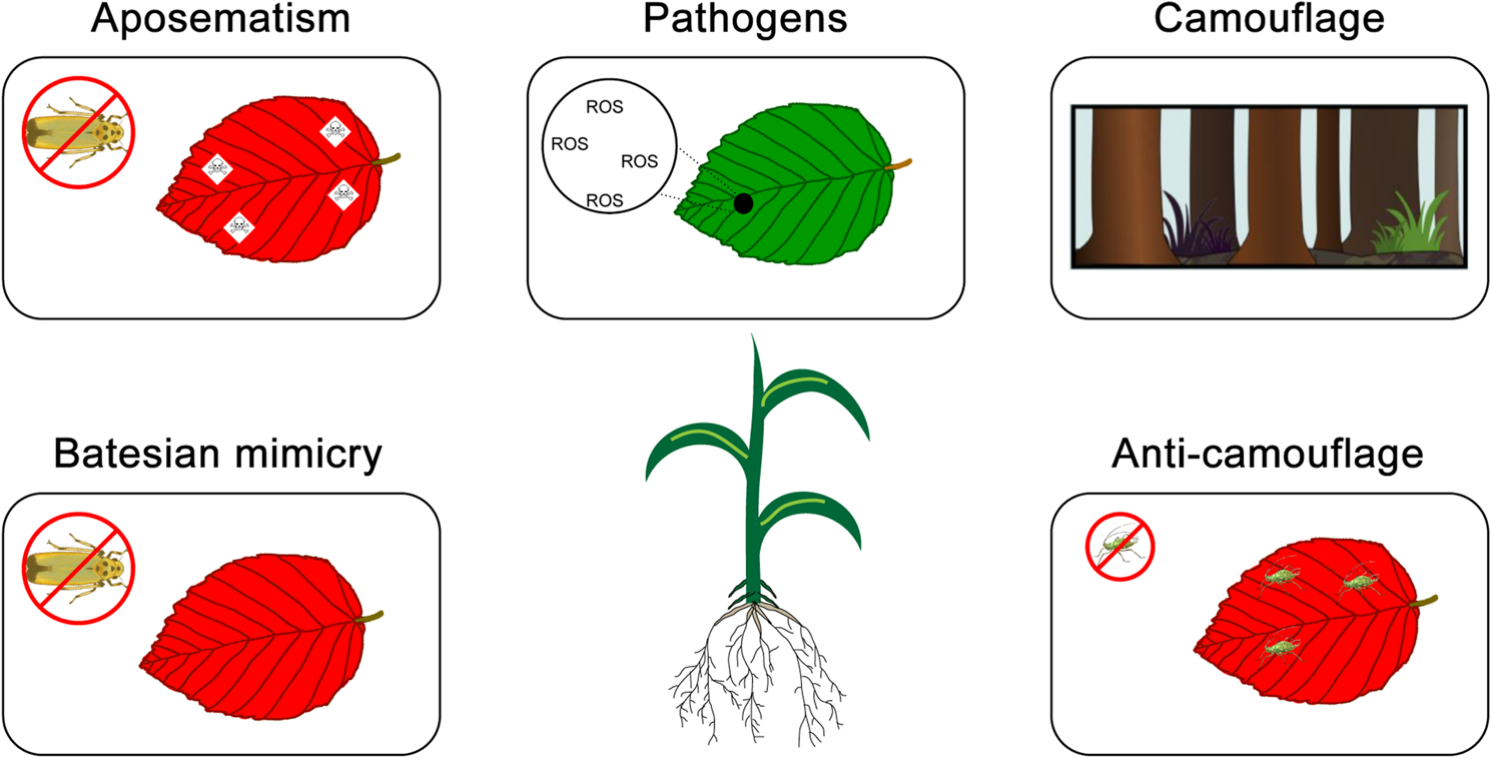
Ecological functions of anthocyanins in plants. Aposematism: Bright coloration signals toxicity or unpalatability, deterring herbivores. Mimicry: Coloration imitates threat for herbivores (e.g. toxicity, unpalatability, or thorns). Pathogens: Anthocyanins have been showed to contribute to pathogen tolerance and resistance, possibly due to their interference with reactive oxygen species (ROS). The exact defense mechanism remains unclear. Camouflage: Dark pigmentation of plants is proposed to protect plants against color-blind herbivores. This part of this figure is based on Wolff & Pucker [271]. Anti-camouflage: Bright coloration can break the crypsis of herbivores, exposing them to predators

Plants are under constant threat by a range of different herbivore and parasite attacks directed against different plant structures and exposed to pathogen infections (Fig. 7). Herbivores might target canopy, low branches, or seedlings while parasites might extract sugar and nitrogen from leaf veins or eat the leaf tissue [299]. As anthocyanins are usually not toxic to animals [300], their role can be considered as minor compared to many other defense compounds [299, 301]. However, the biosynthesis of anthocyanins is tightly connected to the biosynthesis of proanthocyanidins, which have been reported as herbivore repellents [302]. A study investigating tropical trees observed lower herbivore damage on plants with high anthocyanin and tannin content [303]. Similarly, a feeding experiment observed that Helicoverpa zea and Trichoplusia ni larvae ate less of anthocyanin pigmented sectors of petunia flowers compared to white flower segments [304]. The authors further supported the defense role of anthocyanins by demonstrating that anthocyanin extracts can reduce the larva weight gains when added to their diet at approximately natural concentrations [304]. In light of the current knowledge about the coloration functions of anthocyanins, it appears more plausible that the pigments are involved in some kind of visual defense rather than direct repellence. A number of hypotheses have been formulated to explain the role of anthocyanins in defense against herbivores and parasites [299].

Aposematism is the bright coloration of an unpalatable/toxic or thorny plant to scare herbivores. Animals associate the color with an unpleasant experience which prevents or at least reduces future feeding attempts. This has been reported for multiple toxin-rich or unpalatable plant parts including fruits [305], seeds [306, 307], flowers [308, 309], and thorns [310]. Given that multiple independent events of aposematism have been described in the context of anthocyanin pigmentation, an evolutionary benefit is highly likely [299]. Since plants can recover better than animals from losing a part of their structures, an adaptive value is supported by the direct advantage of an individual plant that survives the encounter with a herbivore and lowers the chances of future feeding attempts. This could explain why aposematism spread faster in plants than in animals [299]. There are also anthocyanin-pigmented species like the carnivorous *Drosera spatulata* [311], where anthocyanins might have different functions. Ecological pleiotropy regarding the plant-insect interaction has been suggested in a recent study on green and red color morphs of the carnivorous plant species *Sarracenia purpurea* [312]. Nevertheless, deterrence of herbivores by pigmentation and other means has been proposed as an important first line of plant defense [313].

Bright coloration could also appear as Batesian mimicry i.e. non-toxic species showing bright colors to be mistaken with toxic plants occurring in the same habitat. It was postulated that Batesian mimicry in sessile plants would be less effective than in animals, because herbivores have more time to assess an individual and feeding attempts would not be detrimental for the herbivore [307]. Nevertheless, anthocyanin pigmentation could also contribute to cryptic mimicry by letting a plant structure appear dead or in a senescing stage [314, 315]. The pigmentation could also generate the impression of defensive structures like thorns that are not actually sharp [316]. Since ants are often attracted by plants to defend against herbivores, some plant species display dark spots arranged in a way to mimic a column of ants that would pose a threat to herbivores [299, 317]. Although the pigmentation composition of spots has not been tested, it is believed that they are anthocyanins [299]. Other reports describe aphid mimicry that makes a plant look infested in order to discourage grazers and insects from targeting this plant [317]. Immature pods of several legume species appear to mimic aposematic poisonous caterpillars due to their shape, size, and pigmentation pattern, which might protect these structures against herbivores and also provide an advantage to the entire plant [317]. Multiple functions of anthocyanins turn experimental investigations into a challenge. While high anthocyanin content in some plants could be the result of aposematism or Batesian mimicry, it might just indicate stress in other plant species. A study in *Acer platanoides* observed the highest anthocyanin production and thus red leaves in partially dead trees with the lowest defensive value [318]. Some of these cases, such as ant-like spots or dead-leaf–like pigmentation, are difficult to classify strictly as Batesian mimicry or camouflage, as they may serve both functions depending on context. Camouflage of plant structures due to pigmentation, especially dark pigmentation, has been postulated as another function of anthocyanins [170, 271, 319]. Variegation in understory plants in forests has the potential to disrupt leaf outlines and might protect against color-blind herbivores [319]. The co-occurence of anthocyanins and chlorophyll in seedlings of *Pseudopanax crassifolius* provides the plant with a brownish appearance that resembles the background colors [320]. Anthocyanins could mask the bright green color of chlorophyll-rich leaves thus making them less attractive to certain herbivores [299].

Bright anthocyanin pigmentation could serve as anti-camouflage, i.e., breaking the crypsis of herbivorous insects and making them more visible to predators or parasitoids [321, 322]. Red color of young leaves has been explained as undermining the camouflage of herbivorous insects [322]. This has the potential to deter such herbivores as they might generally avoid surfaces of unsuitable color [299, 322]. This could explain the color differences observed between the adaxial and abaxial side of leaves as well as all other plant structures, because herbivores adapted to the color of one leaf surface would be exposed on all other surfaces [321]. However, this hypothesis is somewhat weakened by a simulation that showed that a visually complex surface has higher potential of hiding insects [323].

Anthocyanins might serve an attraction function in the pollination or seed dispersal, while serving as herbivore defense against other species in the same plant. In *Hypericum calycinum*, dearomatized isoprenylated phloroglucinols, specific derivatives of aromatic polyketides, have an attraction function in the petals, but a repellent function in stamens and ovaries [324]. In *Tipularia discolor*, the same three anthocyanin derivatives stored in histologically different locations result in different leaf colors possibly associated with different functions [25]. It is also feasible that the function of anthocyanins in a plant changes over time [299]. Such synergistic effects could lead to evolutionary advantages thus making plants that use anthocyanins in different ways more likely [325].

Anthocyanins have been reported to contribute to resistances or at least tolerances against pathogens. Heterologous expression of the anthocyanin activator *LEAF COLOUR* from maize in apple resulted in an increased anthocyanin and proanthocyanidin level [326]. A stronger resistance against bacterial infection (*Erwinia amylovora*) and fungal infection (*Venturia inaequalis*) was observed [326]. High concentrations of anthocyanins in purple tomatoes conferred higher tolerance against *Botrytis cinerea* through perturbation of the ROS burst during infection resulting in an extended shelf life [327]. Anthocyanin-rich potatoes have been reported to show better performance against *Pectobacterium carotovorum* when compared to unpigmented potatoes [328]. In summary, multiple studies reported positive effects of high anthocyanin levels against pathogens, but the molecular mechanisms remained sometimes unknown. While an interference with ROS signals associated with infections has been identified as an important and potential universal mechanism [327], it remains feasible that the increased survival of anthocyanin-rich plants is also partially due to better stress resilience due to high levels of antioxidants or the simultaneously increased levels of proanthocyanidins.

## Conclusion

Synthesizing knowledge about anthocyanin biosynthesis across different species, and gaining new insights through comparative analyses of plant lineages, represents a promising direction for future research. The following questions could be addressed using this ‘big data’ approach.

1. How are different decorating reactions contributing to the diversity of anthocyanins? Correlating detected anthocyanins to the presence and activity of decorating enzymes could be a powerful approach if conducted across hundreds or thousands of species. Existing data sets allow this already at the genomics and transcriptomics level.
2. How conserved is the transcriptional regulation of the anthocyanin biosynthesis across plant lineages? Large expression data sets are publicly available for hundreds of plant species which enable investigations of the regulatory networks controlling the anthocyanin biosynthesis.
3. How widespread is independent evolution of steps in the anthocyanin biosynthesis? With the recent interest in the terrestrialization of plants and many genome sequencing efforts committed to early land plants, we can expect to gain a comprehensive understanding of the evolutionary trajectory that resulted in the anthocyanin biosynthesis. Generally, the rapid increase of available plant genome sequences has the potential to reveal cases of independent evolution through comprehensive phylogenies for all genes associated with the anthocyanin biosynthesis.
4. Is there adaptive loss of anthocyanin pigmentation? There are numerous examples of anthocyanin loss within a species or at a genus level. However, reports about anthocyanin loss at the family level are currently restricted to families in the Caryophyllales, where anthocyanins have been replaced by betalains, and Cucurbitaceae, where anthocyanins might have been substituted by carotenoids. More data sets and especially high quality genome sequences facilitate systematic searches for adaptive loss at higher taxonomic levels.
5. How can the anthocyanin biosynthesis be controlled through engineered regulation? Transcriptional regulation is the central level for controlling anthocyanin accumulation and integrating environmental signals. Simultaneously, the transcriptional control poses an elegant system for metabolic engineering in plants. First projects demonstrated the biotechnological potential, but a detailed understanding of nuances in the anthocyanin biosynthesis regulation and especially the role of negative regulators could lead to new targets for engineering via genome editing.
6. What is the relative importance of different anthocyanin functions in a plant lineage? Due to the multifaceted role of anthocyanin functions, this task is similar to solving a system of differential equations. There are already numerous plausible hypotheses about the ecological functions of anthocyanins, but validation on a broad taxonomic level is needed. The relative importance of different anthocyanin functions might vary between plant lineages thus adding another layer of complexity.
7. What is the molecular basis of pigmentation patterns? With the rapid spread of single cell RNA-seq methods, the control of the anthocyanin biosynthesis could be explored at the cell level. Cell-type specific differences in activity of the anthocyanin biosynthesis could be revealed.

## Data Availability

Not applicable.
